# *Micromonas micros* Infection of a Prosthetic Hip Joint: A Case Report and Review of the Literature

**DOI:** 10.1155/2021/9042790

**Published:** 2021-09-20

**Authors:** Abhimanyu Aggarwal, Durane Walker

**Affiliations:** Division of Infectious Diseases, Department of Medicine, University of Massachusetts Medical School, Baystate Medical Center, Springfield, MA, USA

## Abstract

*Micromonas micros* is an oral anaerobic Gram-positive coccus and is a commensal of the mouth, and it is rarely isolated in prosthetic joint infections (PJIs) and even less frequently related to a preceding dental procedure with eventual hematogenous seeding of the prosthetic joint. Here, we present a case of a 56-year-old male with a prosthetic hip joint who developed *Micromonas micros* prosthetic hip joint infection with symptoms starting a few days after a dental procedure and not having received periprocedural antibiotic prophylaxis. He recovered well with surgical intervention and antimicrobial therapy. We conducted a literature review of prosthetic hip joint infections caused by *Micromonas micros* as well as briefly discuss current guidelines on antibiotic prophylaxis in patients with prosthetic joints undergoing dental procedures and some knowledge gaps.

## 1. Case Description

A 56-year-old male was admitted with four days of progressively worsening left hip pain leading to difficulty in ambulation as well as a documented fever of 100.9°F at home. It started immediately after he was working in the yard, and he noted a “popping sensation.” His primary-care provider ordered labs which came back abnormal for leukocytosis 14,000/mm^3^ (predominantly neutrophilic), ESR 66 mm/hr (reference range 0–20 mm/hr) and CRP 12.9 mg/dL (reference range 0–0.5 mg/dL) which prompted the hospital referral. Three days prior to onset of symptoms, he underwent routine dental cleaning with flossing and minor bleeding during the procedure. He was not administered prophylactic antibiotics. He underwent left total hip replacement surgery 5 years prior to this admission and right total hip replacement 8 years prior to this admission, both times for severe osteoarthritis. Other relevant medical history included benign prostatic hyperplasia. He did not indulge in tobacco, alcohol, or illicit drug use. He had no antibiotic allergies.

On admission, his vital signs including temperature were normal. He had a body mass index of 28.9. Pertinent abnormality on examination included extreme difficulty in the range of motion of the left hip due to pain in all directions. Pertinent labs including hemoglobin, platelet counts, and kidney function were unremarkable. X-ray of the left hip ruled out fracture or dislocation. Blood cultures were drawn prior to administration of antibiotics which later returned negative.

He underwent interventional radiology-guided aspiration of the left hip joint on day 2 of hospitalization, with retrieval of 5 cc of pus. Unfortunately, the specimen could not be evaluated for crystals or cell counts since it was described as too viscous. However, Gram staining demonstrated numerous neutrophils and moderate Gram-positive cocci in pairs and chains. Intravenous (IV) vancomycin (2 grams loading dose, followed by 1.5 grams every 12 hourly) and ceftriaxone 2 grams daily were started. On day 3, he was taken to the operating room and underwent total left hip revision with liner swap and irrigation and debridement. Mild joint loosening was noted intraoperatively, and joint fluid cultures were sent.

**Figure 1:**
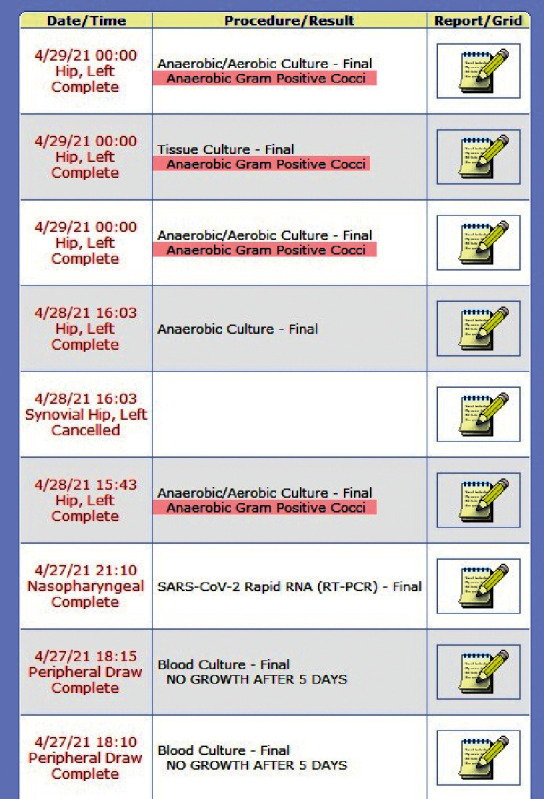
Compiled view of culture results.

**Figure 2:**
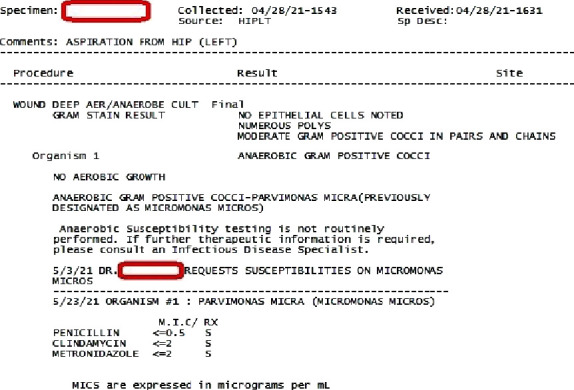
Details of hip aspirate culture results.

**Figure 3:**
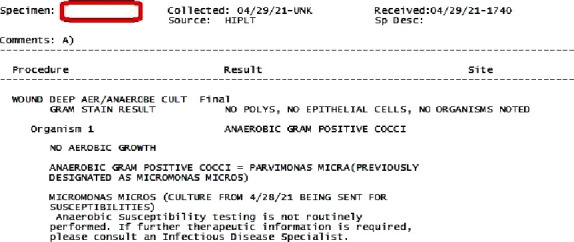
Details of intraoperative culture results.

Specimens from day 2 as well as day 3 came back positive in anaerobic cultures for *Micromonas micros,* susceptible to penicillin, clindamycin, and metronidazole (Figures 1–3). A diagnosis of left hip prosthetic joint infection with debridement, antibiotics, and implant retention (DAIR) was established. Vancomycin was discontinued, and he was discharged on IV ceftriaxone 2 grams daily via peripherally inserted central venous catheter line for 6 weeks for ease of dosing. Weekly follow-up labs were performed and demonstrated normalization of WBC and inflammatory markers (ESR and CRP). Overall, he clinically improved and was later transitioned to 6 weeks of oral penicillin V 1 gram three times a day.

## 2. Discussion

*Micromonas micros* (*M. micros*), which has now been renamed *Parvimonas micra* and previously called *Peptostreptococcus micros*, is an anaerobic Gram-positive coccus, usually a member of oral flora and often has been isolated from the respiratory tract and gastrointestinal tract, as well as the female genital tract [7, 8]. Anaerobic oral micro-organisms have been implicated as the causative agents for late-onset prosthetic joint infections (late in reference from the time of prosthetic joint creation) [1].

*M. micros* has been infrequently reported as a cause of prosthetic joint infections. Randall et al. reviewed the literature for *M. micros* prosthetic joint infection of the knee joint and included 7 cases [9]. Overall, it was noted that different authors used different antibiotics for management and reported varying time duration between initial prosthetic knee joint creation and onset of symptoms of knee PJI. Only 5 of these cases had a documented preceding dental procedure. Here, we reviewed the literature for all the reported hip joint PJI's with *M. micros* and found 10 reported cases with scant information about several aspects of management, as reported in Table 1. However, like our case, only the case report by Bartz et al. reported a potential association between the preceding dental procedure and isolation of *M. micros* in the hip PJI [2]. Another study from 2001 reported 4 cases of *P. micros* isolated in late-onset orthopedic implant infections but did not specify the site for 2 of them as well as the treatment outcome in all 4 [10].

For nearly four decades, a controversy has existed around the decision on antibiotic prophylaxis for patients undergoing dental procedures who also have prosthetic joints. Despite the existing evidence of transient bacteremia following dental procedures [11, 12], guidelines still advocate against the use of prophylactic antibiotic prior to proceeding with a dental procedure in patients with prosthetic joints. The American Academy of Orthopedic Surgeons, American Dental Association, and Infectious Diseases Society of America [13–15] in their guidelines attributed to lack of evidence to associate a dental procedure with a prosthetic joint infection. However, they do make an exception for patients who are immunosuppressed (for example, malignancy or diabetes mellitus) or are on immunosuppressive therapy or have a prior history of prosthetic joint infection.

An important point was raised by Moreira et al. in a systematic review of literature for antibiotic prophylaxis for patients with prosthetic joints undergoing dental procedures [16]. Studies reviewed to come to the current consensus lacked enough knowledge on the periodontal conditions of the patients included, as well as significant heterogeneity in objective definitions of periodontal diseases. To arrive at a judgement on using antibiotic prophylaxis might require assessment of periodontal health by dental surgeons at the time of their procedure and make a final decision in that moment. This just reinforces the role for the dental surgeons to ensure review of detailed past medical history including the presence of a prosthetic joint, the timing of its creation, and if there is a prior history of PJI.

There were two major reasons why we chose to share this case report with the readers. One was to share literature review of *M. micros* hip PJI. The second was to indulge in thought-provoking discussions about the consensus on the use of antibiotic prophylaxis for patients with prosthetic joints who are about to undergo dental procedures.

In conclusion, *M. micros* hip PJIs are infrequent but still should be included in the list of pathogens responsible for late-onset PJIs. The suspicion for *M. micros* should be higher in patients who undergo dental procedures that put them at risk of bleeding, especially if they also have concurrent periodontal disease. Additionally, the outlook towards antibiotic prophylaxis prior to dental procedures in patients with a known prosthetic joint may possibly reach a consensus if future studies include periodontal condition as a factor affecting the decision-making process. The issue with this strategy is that the dentist would have to know the condition of the teeth and gums prior to prescribing the antibiotics.

## Figures and Tables

**Table 1:** Summary of reported cases of *Micromonas micros* PJI in the hip joint.

Report	Age/gender	Time interval between initial prosthetic joint creation and symptom onset	Antibiotic choice; duration	Outcome
Petrini et al. ^*∗∗∗*^[1]	Male^*∗*^	4 years	—	—
Petrini et al. [1]	Male^*∗*^	3 years	—	—
Petrini et al. ^*∗∗∗*^[1]	Male^*∗*^	3 years	—	—
Petrini et al. ^*∗∗∗*^[1]	Male^*∗*^	2 years	—	—
Petrini et al. ^*∗∗∗*^[1]	Female^*∗*^	5 years	—	—
Bartz et al. [2]	63 years/female	9 years	IV cefuroxime/gentamicin; clindamycin^*∗∗*^	Recovered
Bohra et al. [3]	67 years/female	8 months	IV vancomycin 2 weeks	Recovered
Huang et al. [4]	65 years/female	7 years	IV piperacillin/tazobactam 2 weeks; PO amoxicillin/clavulanic acid 8 weeks	Recovered
Marmor et al. [5]	—	—	—	—
Rieber et al. [6]	78 years/female	6 months	—	—
Our case report, 2020	56 years/male	5 years	IV ceftriaxone 6 weeks; PO penicillin V 6 weeks	Recovered

All patients had surgical intervention done for the PJI, except for lack of data on Marmor et al.'s case. ^*∗*^Age not specified, ^*∗∗*^duration not specified, ^*∗∗∗*^*M. micros* isolated as a part of polymicrobial infection IV, intravenous; PO, per oral.

## Data Availability

No data were used to support the study.
